# A Successful Endovascular Technique for Complete False Lumen Thrombosis in Chronic Abdominal Aortic Dissection

**DOI:** 10.3400/avd.cr.20-00163

**Published:** 2021-03-25

**Authors:** Hiromitsu Hiruma, Yukihisa Ogawa, Kiyoshi Chiba, Takaaki Maruhashi, Akiyuki Kotoku, Hidefumi Mimura, Takeshi Miyairi, Hiroshi Nishimaki

**Affiliations:** 1 Department of Radiology, St. Marianna University School of Medicine, Kawasaki, Kanagawa, Japan; 2 Department of Cardiovascular Surgery, St. Marianna University School of Medicine, Kawasaki, Kanagawa, Japan; 3 Department of Emergency and Critical Care Medicine, Kitasato University School of Medicine, Sagamihara, Kanagawa, Japan

**Keywords:** EVAR, abdominal aneurysmal dilation, chronic aortic dissection

## Abstract

A 66-year-old man presented with an enlarging abdominal aorta false lumen, after type A aortic dissection repair. Residual entries were located at the left renal artery, abdominal aorta, and left external iliac artery. The patient underwent endovascular aortic repair with left renal artery stenting to close the entries. Completion aortography showed no false lumen flow without an endoleak, and contrast-enhanced computed tomography 1 month after the procedure demonstrated complete false lumen thrombosis. A total endovascular approach is possible for abdominal aneurysmal dilation in chronic aortic dissection when all entries can be closed using a one-stage procedure with stent grafts and/or branch stenting.

## Introduction

Despite improvements in the treatment of acute type A aortic dissection, several complications, such as aneurysmal false lumen dilation and rupture, have been reported. Evangelista et al. reported that 9% of patients with surgically treated type A aortic dissection require further therapy during follow-up because of descending aortic dilation.^1)^ Although there are several reports on the usefulness of fenestrated/branched endovascular aortic repair (EVAR) for chronic aortic dissection,^2,3)^ these techniques have not been approved in Japan. Therefore, we report complete false lumen thrombosis (FLT) of abdominal aortic dissection, followed by EVAR with branch stenting as a one-stage procedure for chronic aortic dissection.

## Case Report

**Figure 1:**
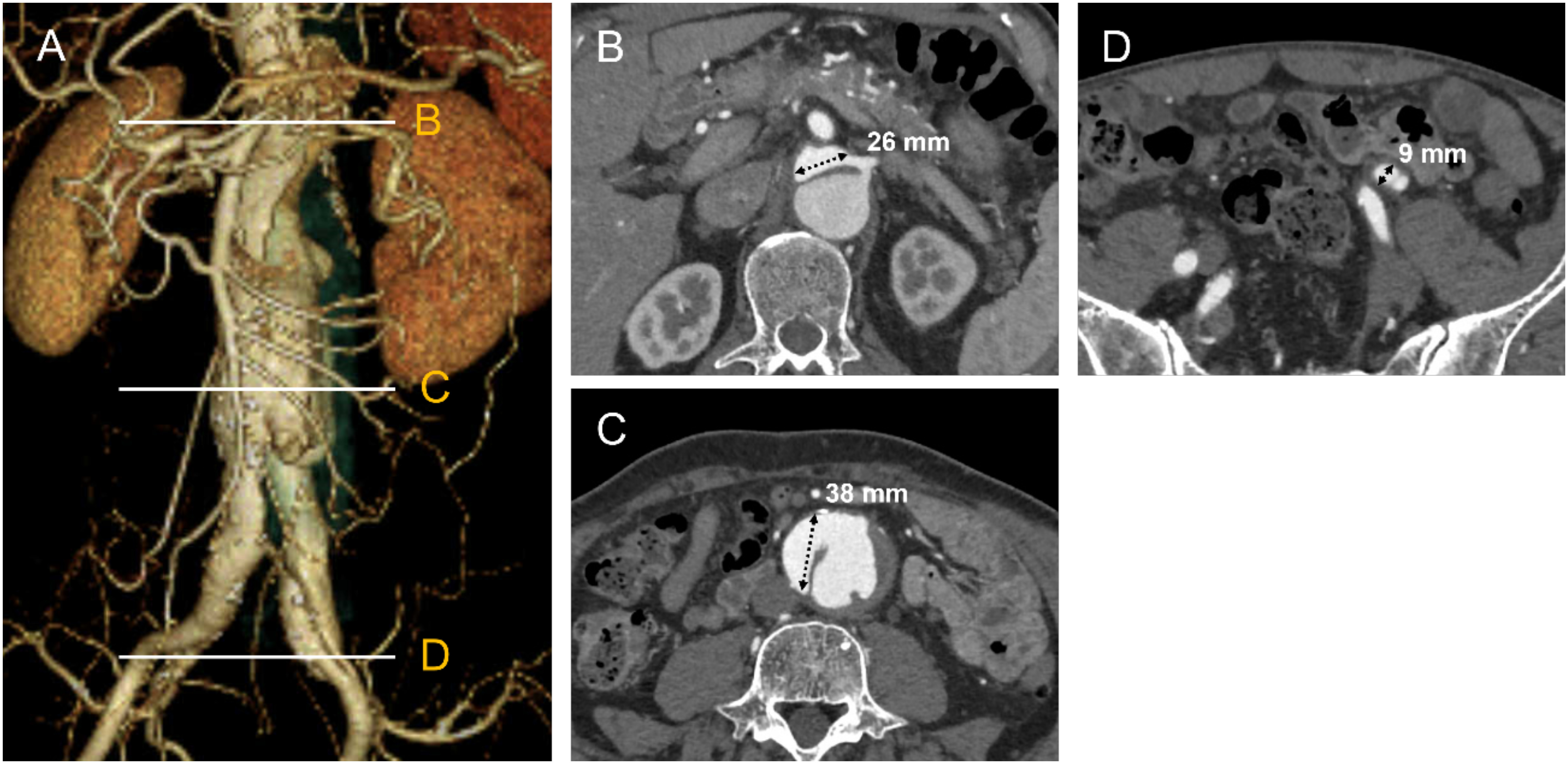
Fig. 1 Preoperative volume rendering showing the false lumen flow from the proximal abdominal aorta to the left external iliac artery (EIA) (**A**). Contrast-enhanced computed tomography axial images in the early phase showing the entries at the left renal artery orifice (**B**), descending aorta (**C**), and the left EIA (**D**).

**Figure 2:**
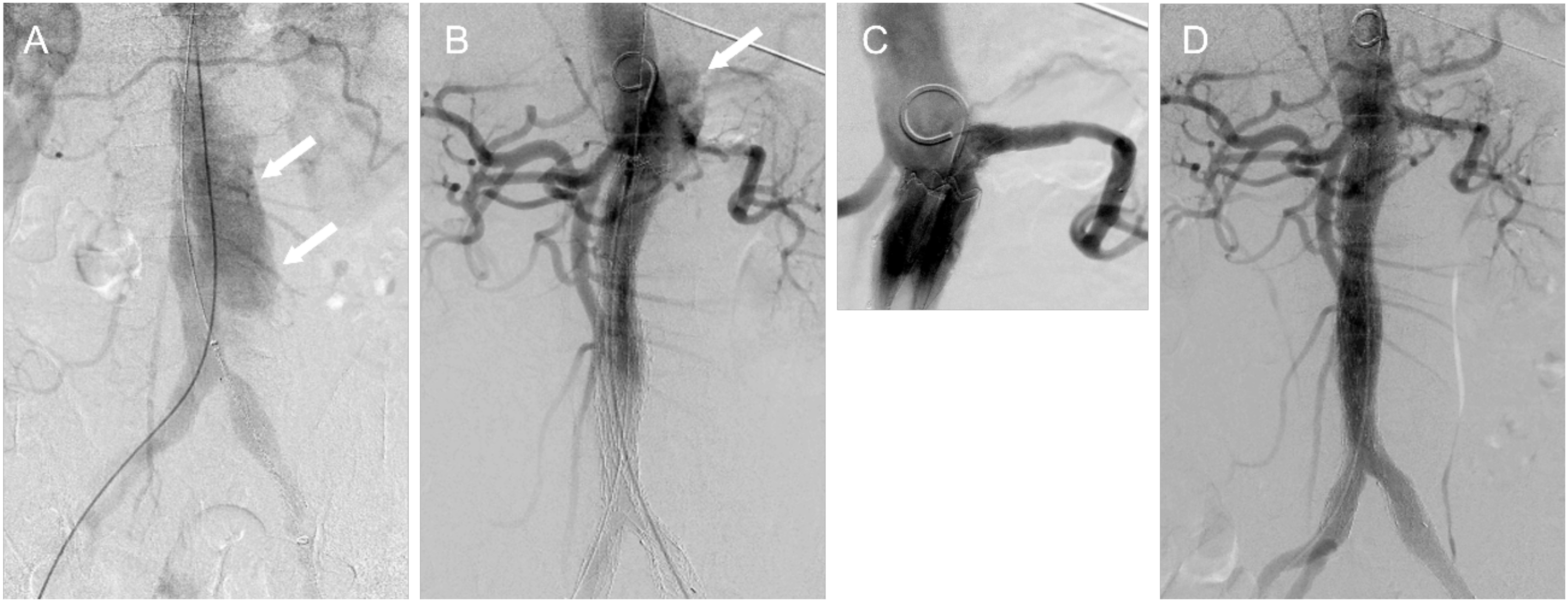
Fig. 2 Aortography images showing the large false lumen (FL) (**A**; arrows). Residual entry in the left renal artery after placing the AFX device (**B**; arrow). No flow into the FL in the left renal artery is seen after placing the VBX (**C**). Completion aortography demonstrating no FL flow without endoleaks (**D**).

**Figure 3:**
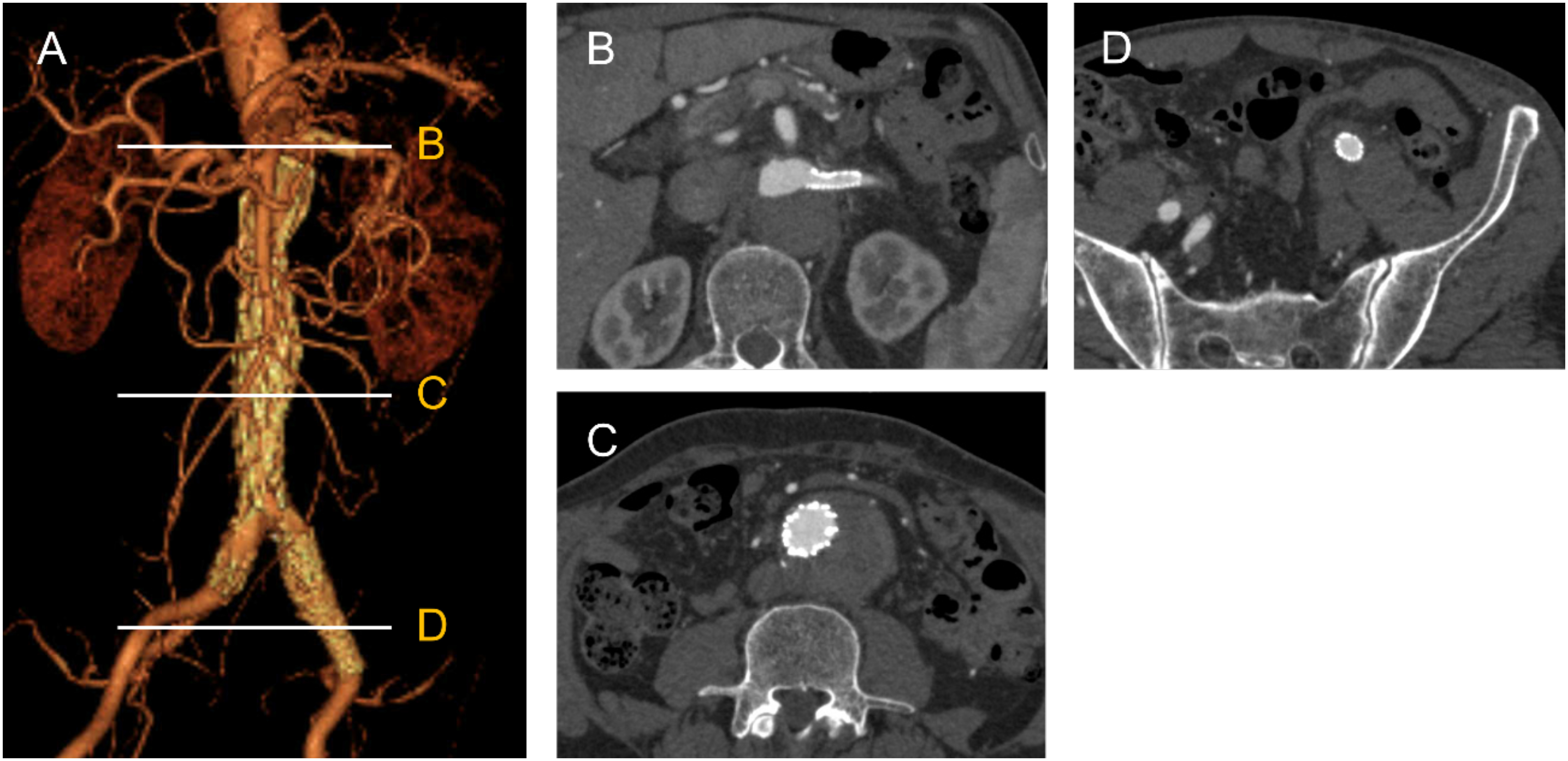
Fig. 3 Contrast-enhanced computed tomography 1 month after the procedure showing no false lumen flow without endoleaks (**A**–**D**).

**Figure 4:**
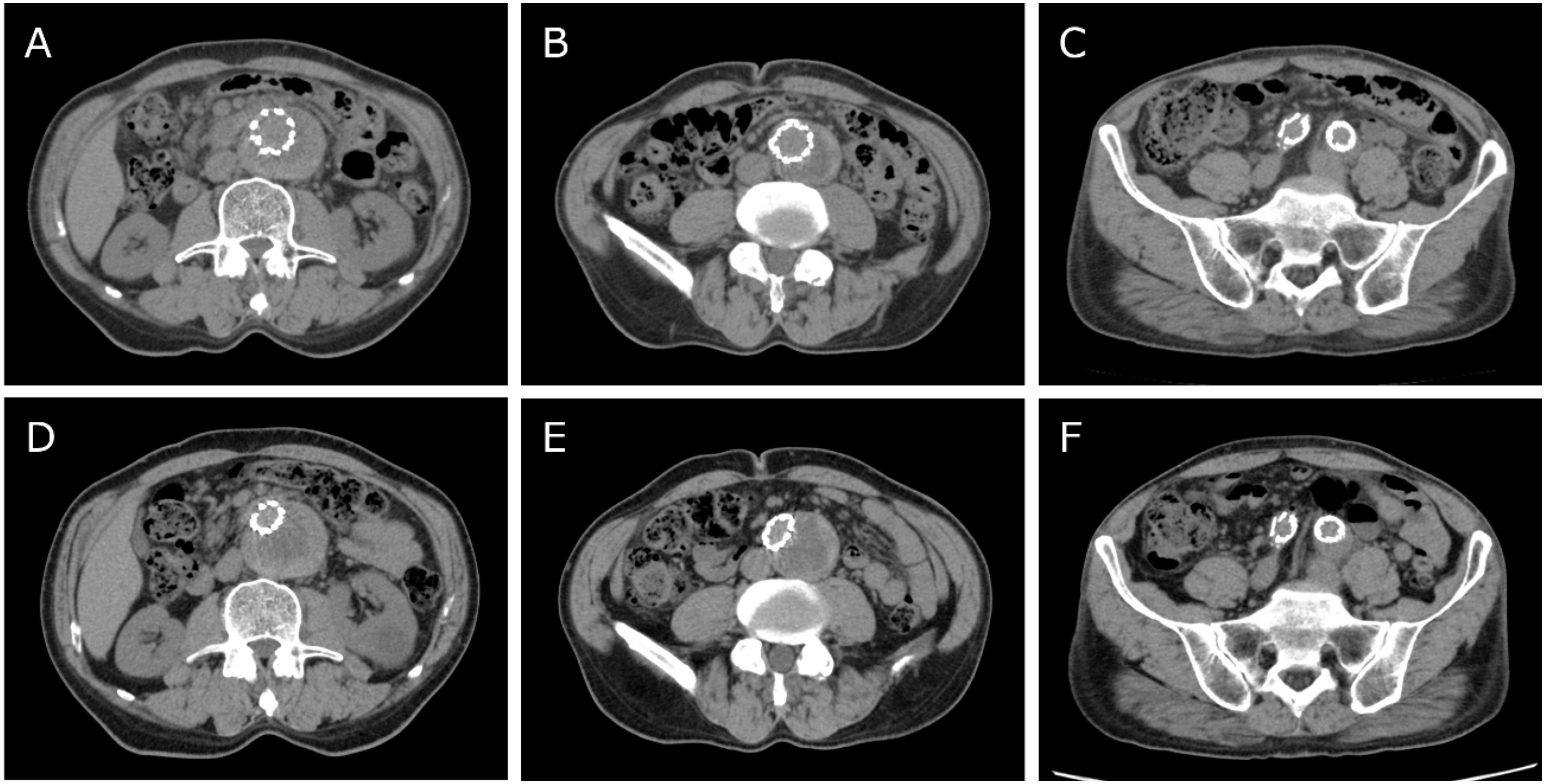
Fig. 4 Plain computed tomography (CT) 7 months after the procedure showing a slight reduction in the diameter of the abdominal aorta and good expansion of the stent graft (**A**, **B**, **C**) compared with the same level of CT 1 month after the procedure (**D**, **E**, **F**).

A 66-year-old man underwent type A aortic repair 14 years prior to presentation. He had an enlarging false lumen (diameter, 53 mm) in the abdominal aorta. Contrast-enhanced computed tomography (CT) showed chronic communicating aortic dissection beginning in the distal descending aorta and extending into the left external iliac artery (EIA). Residual entries were located at the orifice of the left renal artery, infrarenal abdominal aorta, and left EIA (**Fig. 1**). EVAR with branch stenting was scheduled to close all residual entries. This procedure was performed in a hybrid operating room using an Infinix Celeve™-i (Toshiba Medical, Tochigi, Japan) under general anesthesia. First, we placed a 16 mm×12 mm×7 cm Excluder extension (W. L. Gore & Associates, Inc., Flagstaff, AZ, USA) in the left EIA via a 12-F left femoral Gore DrySeal sheath (W. L. Gore & Associates). Second, we performed EVAR using AFX devices (25 mm×95 mm aortic cuff and 22 mm×[90+30]×20 mm; Endologix, Irvine, CA, USA). Then, we placed a 7 mm×29 mm VBX balloon-expandable endoprosthesis (W. L. Gore & Associates) in the left renal artery. Completion aortography showed no false lumen flow without an endoleak (**Fig. 2**), and contrast-enhanced CT 1 month after the procedure revealed complete FLT (**Fig. 3**), and plain CT 7 months after the procedure showed a slight reduction in the diameter of the abdominal aorta and good expansion of the stent graft (**Fig. 4**).

## Discussion

This case provided two important clinical suggestions. Closing all residual entries using a one-stage procedure with stent grafts and/or branch stenting can achieve complete FLT for enlarging abdominal aneurysmal dilation in chronic aortic dissection. AFX devices with a unibody design may be suitable for this approach, because narrowing of the true lumen often terminates at the distal end of the abdominal aorta.

There are significantly higher risks of reintervention and aneurysm rupture in chronic aortic dissection after thoracic EVAR/EVAR than after open abdominal aortic aneurysm repair owing to reperfusion from distal entry tears or from visceral branch vessels.^4,5)^ Accordingly, it is important to promote complete FLT, as this is associated with a better long-term prognosis.^6)^ Kanaoka et al. reported that complete FLT in chronic aneurysmal aortic dissection was achieved in 54.8% of patients in the complete exclusion group, in which all entry and reentry sites were closed, and in 2.3% of patients in the partial exclusion group, in which the entry site alone was closed.^7)^ Attempting to close all residual entries can effectively lead to complete FLT and decrease reinterventions and aortic-related complications. Therefore, in our case, we attempted to close all entries in a one-stage procedure using various devices and eventually achieved complete FLT.

AFX devices with a unibody design are bifurcated endografts that provide anatomical fixation to prevent migration when positioned directly on the aortic terminus. These devices can be used in combination with iliac limb extensions.^8)^ We used the AFX combined with the Excluder extension because there was narrowing of the terminal aorta, and one of the residual entries was in the left EIA. One possible concern was the stent graft-induced new entry in the proximal landing zone due to excessive oversizing. Therefore, we decided that the size of the stent graft should be nearly equal to the flap length of the proximal landing zone to avoid flap injury.

We placed the VBX endoprosthesis into the left renal artery to bridge the intimal opening and the branch and flared the VBX proximally to prevent an endoleak. Fenestrated/branched devices may be an option, but these are not available in Japan. Furthermore, chronic aortic dissection often has a narrowing true lumen that makes fenestrated/branched EVAR difficult.^9)^

Oikonomou et al. reported that 47.4% of patients with fenestrated/branched stent graft post-dissection thoraco-abdominal aneurysm required reintervention within 3 years.^2)^ The main reasons for reintervention were endoleaks from visceral/renal arteries and iliac endoleaks. The present case is also at a risk of an endoleak through visceral arteries branching from the false lumen. Prophylactic embolization of these branches may solve this issue, but vessel or flap injury should be avoided during the procedure. Although this patient showed no endoleak for at least half a year, this should be monitored closely.

## Conclusion

EVAR using the AFX device with branch stenting as a one-stage procedure can achieve complete FLT of large abdominal aortic aneurysmal dilation in chronic aortic dissection.

## Additional Note

Written informed consent was obtained from the patient for publication of the case details.

## References

[1] Evangelista A, Salas A, Ribera A, et al. Long-term outcome of aortic dissection with patent false lumen: predictive role of entry tear size and location. Circulation 2012; 125: 3133-41. 22615344 10.1161/CIRCULATIONAHA.111.090266

[2] Oikonomou K, Kasprzak P, Katsargyris A, et al. Mid-term results of fenestrated/branched stent grafting to treat post-dissection thoraco-abdominal aneurysms. Eur J Vasc Endovasc Surg 2019; 57: 102-9. 30181064 10.1016/j.ejvs.2018.07.032

[3] Marques De Marino P, Ibraheem A, Gafur N, et al. Outcomes of fenestrated and branched endovascular aortic repair for chronic post-dissection thoracoabdominal aortic aneurysms. J Cardiovasc Surg (Torino) 2020; 61: 427-34. 10.23736/S0021-9509.20.11367-3 32319276

[4] Stather PW, Sidloff D, Dattani N, et al. Systematic review and meta-analysis of the early and late outcomes of open and endovascular repair of abdominal aortic aneurysm. Br J Surg 2013; 100: 863-72. 23475697 10.1002/bjs.9101

[5] Boufi M, Patterson BO, Grima MJ, et al. Systematic review of reintervention after thoracic endovascular repair for chronic type B dissection. Ann Thorac Surg 2017; 103: 1992-2004. 28434549 10.1016/j.athoracsur.2016.12.036

[6] Wojtaszek M, Wnuk E, Maciag R, et al. Promoting false-lumen thrombosis after thoracic endovascular aneurysm repair in type B aortic dissection by selectively excluding false-lumen distal entry tears. J Vasc Interv Radiol 2017; 28: 168-75. 27645464 10.1016/j.jvir.2016.07.007

[7] Kanaoka Y, Ohki T, Kurosawa K, et al. Early and midterm outcomes of endovascular treatment for chronic aneurysmal aortic dissection: a retrospective study. Ther Adv Cardiovasc Dis 2018; 12: 275-87. 30071800 10.1177/1753944718792453 PMC6120181

[8] Welborn MB 3rd, McDaniel HB, Johnson RC, et al. Clinical outcome of an extended proximal seal zone with the AFX endovascular aortic aneurysm system. J Vasc Surg 2014; 60: 876-83; discussion, 883-4. 24877852 10.1016/j.jvs.2014.04.017

[9] Oikonomou K, Katsargyris A, Ritter W, et al. Endovascular management of chronic post-dissection aneurysms. Ann Cardiothorac Surg 2014; 3: 307-13. 24967171 10.3978/j.issn.2225-319X.2014.04.02 PMC4052415

